# Comparative Analysis of High-Torque-Density Permanent Magnet Motors Having Similar Slot and Pole Numbers for Humanoid Robot Applications

**DOI:** 10.3390/biomimetics11060412

**Published:** 2026-06-11

**Authors:** Kun Bi, Zhuoyi Chen, Tianran He

**Affiliations:** 1 School of Machinery and Transportation, Changzhou Vocational Institute of Industry Technology, Changzhou 213164, China; bk@ciit.edu.cn; 2 School of Information Science and Engineering, NingboTech University, Ningbo 315100, China; 3 School of Electronic and Information Engineering, Tongji University, Shanghai 200092, China; hetianran@tongji.edu.cn

**Keywords:** high-torque-density permanent magnet motor, external rotor, slot/pole combination, overload capability

## Abstract

The conventional robotic position control is gradually being replaced by force control, which is commonly used in humanoid robot applications that require force interaction with the environment, force transmission, or contact. A high-back-drive-efficiency actuator with a high-torque-density permanent magnet motor connecting the low-ratio planetary reducer is widely applied in interactive robotic systems without a torque/force sensor. This paper proposes a high-torque-density permanent magnet motor with an external rotor structure, which can realize torque enhancement by the increased air-gap diameter and better space utilization by the internal planetary reducer, i.e., the reducer inside the stator. First, the motor topologies with different slot/pole number combinations are introduced. Then, the optimization of a slot/pole number combination is elaborated for the maximum torque and torque mass density. In addition, the influence of the slot/pole number combination on the torque characteristic and overload capability is investigated by the finite element (FE) method. The experimental results of the prototype motor are provided to verify the analysis.

## 1. Introduction

The evolutionary trajectory of humanoid robots has witnessed a significant transformation in core actuation systems: a gradual transition from early-stage electromechanical assemblies to today’s highly dynamic, electric-driven platforms. Honda’s ASIMO robot, introduced in 2000, established a pioneering milestone in the realm of bipedal locomotion; it utilized traditional servo motors paired with harmonic drives to achieve stable walking, albeit with relatively limited dynamic agility [1,2,3]. Subsequently, Boston Dynamics’ Atlas robot demonstrated unprecedented athletic capabilities through the use of hydraulic actuation technology [4,5,6]. By leveraging the high-power density inherent in fluid power systems, it successfully executed a variety of complex acrobatic feats, including backflips and parkour maneuvers. However, the inherent drawbacks of hydraulic systems—including excessive noise, complex maintenance requirements, and low energy efficiency—precipitated a paradigm shift across the industry, driving a transition toward fully electric actuation systems. The latest generation of humanoid robots—exemplified by Tesla’s Optimus [7,8,9] and MIT cheetah [10,11,12,13]—has fully embraced integrated electromechanical actuator modules; these modules combine high-performance permanent magnet synchronous motors (PMSMs), precision torque sensors, and advanced planetary or cycloidal gear reducers. This technological transition not only endows these robots with dynamic performance comparable to that of hydraulic systems but also provides them with intrinsic force feedback and impedance control capabilities; this enables the robots to engage in compliant physical interactions within unstructured environments while simultaneously ensuring manufacturability and cost-scalability advantages for commercial deployment.

Due to the force control requirements of humanoid robots, quasi-direct drive (QDD) actuators with high force transmission transparency are needed [14,15]. These actuators enable force feedback based on current sampling variations without the need for an additional force sensor. A quasi-direct drive actuator consists of three components: a planetary gearbox with a low reduction ratio (<20:1), a high-torque-density permanent magnet motor, and a high-power-density driver. For high-torque-density permanent magnet motors, both external rotor [16] and internal rotor [17,18,19] surface-mounted permanent magnet motors (ERSPMs and IRSPMs) have been compared and employed for industrial applications. However, different rotor structures exhibit distinct characteristics. The internal rotor motor offers higher response speed and provides strong winding cooling capability when combined with a housing featuring a thermal dissipation structure. In contrast, the external rotor motor has a larger air-gap diameter, offering an absolute advantage in electromagnetic performance with a high torque coefficient. Additionally, the cup-shaped rotor possesses a relatively large inertia, which contributes to stable rotor operation. In the current context where force control capability is widely demanded for humanoid robots, planetary gearboxes with low reduction ratios provide favorable back drivability, enabling sensorless force feedback. Nevertheless, high torque output remains a critical requirement for humanoid robot joints. Therefore, compared with internal rotor motors, external rotor motors are more suitable for integration with low-ratio planetary gearboxes to form high-power-density actuators [20].

For external rotor permanent magnet motors, the optimal split ratio has been studied by analytical and FE methods [21,22], and the comparison of the overlapping and non-overlapping windings is investigated in [23]. For the joint applications, the 36-slot/42-pole ERSPM has been employed in legged robots [24,25,26]; however, the 18-slot/20-pole ERSPM has been used in a hip exoskeleton [27], which is the same combination as the 36-slot/40-pole [28]. compares the 24-slot/22-pole and 18-slot/20-pole ERSPMs for exoskeleton joint integration. However, few papers compare the electromagnetic performance of the motors with different slot/pole combinations, especially for the overload capability. Therefore, this paper will optimize the 24-slot ERSPMs with different pole numbers and compare the torque characteristics and overload capability. In addition, the primary contribution of this work lies in the systematic design of the wrist actuator tailored to humanoid robot requirements, coupled with the optimization and comparative analysis of multi-pole motor configurations. Additionally, the thermal analysis demonstrates the motor’s temperature performance under various operating conditions, thereby validating the feasibility of the electromagnetic design.

In this paper, Section 2 shows the motor topologies of the 24-slot ERSPMs with different pole numbers. The optimization of the 24-slot/28-pole ERSPM is described in Section 3, and with the same optimized stator parameters, the different pole numbers are compared in Section 4. Four 24-slot motors with different pole numbers are optimized by the FE method and the influence of the pole number on the optimal results and torque is investigated in Section 5. Section 6 is the conclusion.

## 2. Actuator Requirements and Motor Topology

**Figure 1:**
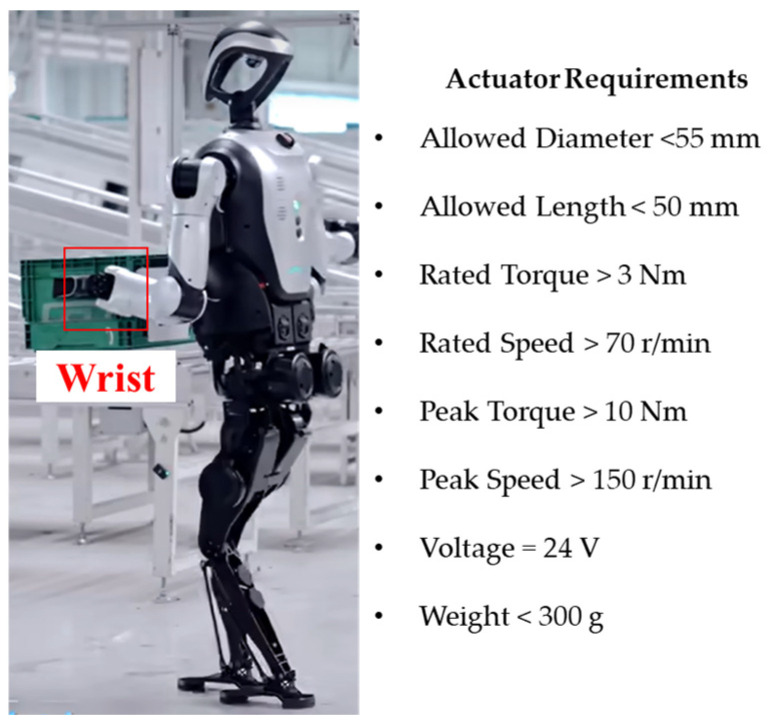
KUAVO humanoid robot and wrist actuator requirements.

Humanoid robots, featuring dual manipulators and articulated end-effectors, exhibit considerable promise for deployment in domestic assistance and manufacturing settings. A typical humanoid robot, KUAVO from Leju (Shenzhen City, China) Robotics Co., Ltd., is shown in Figure 1, and it can handle box transportation in an industrial scenario. This operating condition imposes specific requirements on the wrist actuator, as illustrated in Figure 1.

**Figure 2:**
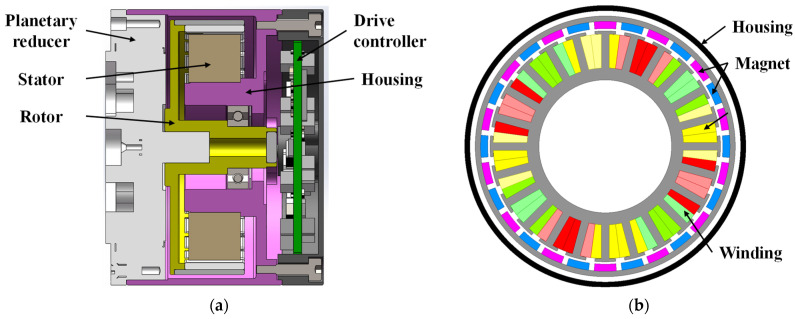
Actuator and motor topologies. (**a**) Actuator with a 10:1 planetary reducer, a PM motor, and a drive controller; (**b**) 24-slot/28-pole ERSPM.

Since the proposed motor is designed for the wrist joint in the humanoid robot arms, the joint size is limited, which means the housing diameter of the actuator is fixed. The cross-sectional view of the actuator based on the proposed 24-slot/28-pole ERSPM is shown in Figure 2a, and the motor topology is shown in Figure 2b. It can be seen that the actuator includes a 10:1 planetary reducer, a PM motor, and a drive controller. To enhance the heat dissipation capability, the planetary reducer adopts an external connection configuration with respect to the motor, and the stator is in an interference fit with the housing, as shown in Figure 2a. In addition, there are two air-gaps in the actuator, one between the housing and rotor, and the other one between the stator and rotor. Based on the mechanical design and manufacturing constraints, the housing diameter is 53 mm, the housing thickness is 1 mm and the outer air-gap length is also 1 mm, which means the maximum rotor diameter is 49 mm. In Figure 2b, the different colors in the stator mean the three-phase windings, and the dark and light means the positive and negative currents. Two colors in the magnet mean N- and S-pole.The main parameters of the proposed motor are shown in Table 1.

## 3. Parameter Optimization

The 24-slot/28-pole ERSPM is optimized by the finite element (FE) method. With a fixed copper loss for the same temperature rise, the optimal goals are the output torque and torque mass density due to the lightweighting design. In general, the torque increases with the decrease in the rotor yoke thickness due to the larger air-gap diameter. However, considering the challenges in mechanical machining, the rotor thickness is designed as 1.0 mm. Since the fundamental design of a 24-slot/28-pole ERSPM is established, the variables should be designed to enhance the electromagnetic torque. For conventional PM synchronous motors, the average torque can be calculated by [22]:

(1) $$T=3\pi \alpha_{p}B_{g}L_{a}D_{a}N_{a}I_{rms}/2\sqrt{2}$$

where *α_p_* represents the pole-arc coefficient, *B_g_* is the average air-gap flux density, *L_a_* is the stator active length, *D_a_* is the stator outer diameter, *N_a_* is the number of turns per, and *I_rms_* is the root-mean-square (RMS) phase current.

For PM synchronous motors, copper loss generally constitutes the dominant loss component. Therefore, assuming fixed copper loss (*P_cu_*)—which implies constant temperature rise for a given motor size—and maintaining a constant slot fill factor, the RMS phase current can be expressed in terms of copper loss as

(2) $$I_{rms}=\sqrt{\frac{P_{cu}k_{a}S_{slot}}{36{N_{a}}^{2}\rho (L_{a}+L_{end})}}$$

where *k_a_* represents the slot fill factor; *S_slot_* is the total slot area; *L_end_* denotes the end-winding length of windings; and *ρ* represents the electrical resistivity.

Therefore, replacing the phase current by Equation (4), the torque can be rewritten as

(3) $$T=\frac{3\pi }{2\sqrt{2}}\alpha_{p}L_{a}(B_{g})(\lambda D_{ro})\sqrt{\frac{P_{cu}k_{a}S_{slot}}{36\rho (L_{a}+L_{end})}}$$

where *λ* is the split ratio, i.e., the ratio of the stator outer diameter and the rotor outer diameter. *D_ro_* is the rotor outer diameter.

(4) $$B_{g}=\frac{2}{\pi }B_{\delta };\;B_{\delta }=\frac{B_{r}}{1+\frac{u_{r}l_{g}}{l_{m}}}$$

(5) $$B_{m}=\frac{\tau_{s}}{w_{t}}B_{g};\;B_{m}=\frac{\tau_{p}}{2h_{y}}B_{g}$$

where *B_r_* is the remanence of the magnet, *u_r_* is the relative permeability, *l_g_* is the air-gap length, *l_m_* is the magnet thickness, and *B_m_* is the maximum flux density of the stator tooth. *Τ_s_* is the slot pitch, *w_t_* is the stator tooth width, *Τ_p_* is the pole pitch, and *h_y_* is the stator yoke height.

It can be seen that when a split ratio (*λ*) is given, the magnet thickness (*l_m_*) can be calculated due to the fixed rotor outer diameter and rotor yoke thickness. Then, the air-gap flux density (*Bg*) can be obtained with a fixed air-gap length (*l_g_*). In addition, when a maximum flux density (*B_m_*) is given, the stator tooth width and yoke thickness can be calculated, and the slot area is a result, as well as the torque. Therefore, the main variables in this optimization include the split ratio (*λ*), i.e., the ratio of the stator outer diameter to the rotor outer diameter, and the maximum stator flux density (*B_m_*).

With a given *λ*, the magnet thickness can be calculated, and the air-gap flux density can be obtained. Meanwhile, the stator tooth width and stator yoke thickness can be calculated by a given *B_m_*, and thus the specific slot area. With a fixed slot fill factor, the wire area and resistance can be obtained, which leads to the RMS phase current and output torque under the fixed copper loss.

**Figure 3:**
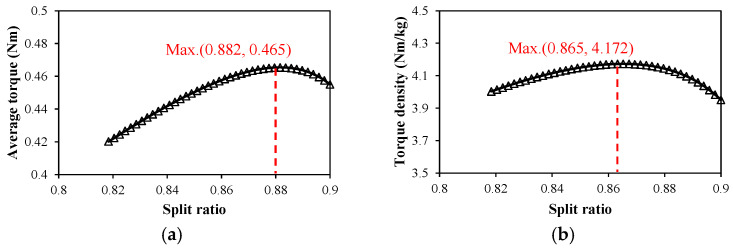
Optimal results of the proposed motor. (**a**) Variation in the average torque with the split ratio. (**b**) Variation in the torque mass density with the split ratio.

Figure 3a shows the variation in the average torque with the split ratio under a given *B_m_* (1.8 T). It can be seen that the torque increases at first and then decreases with the increase in *λ*. When the split ratio is 0.882, the maximum torque can be achieved, i.e., *T_max_* = 0.465 Nm. Figure 3b shows the variation in the torque mass density with the split ratio under a given *B_m_* (1.8 T). It can be seen that the torque density increases at first and then decreases with the increase in *λ*. When the split ratio is 0.865, the maximum torque density can be achieved, i.e., *TD_max_* = 4.172 Nm/kg.

Table 2 shows the parameters of two optimized motors for different optimal goals, named as Motor A and Motor B. It is worth noting that although the two optimal motors, respectively, have the maximum torque and torque density, the difference in the maximum torque between Motor A and B is less than 5%, as well as the torque density. However, Motor B has a larger PM volume than Motor A, and the PM utilization (torque per PM volume) of Motor A is 26.8% higher than that of Motor B. Therefore, Motor A is selected for the electromagnetic performance comparison of different pole numbers.

## 4. Electromagnetic Performance Comparison with the Same Stator

**Figure 4:**
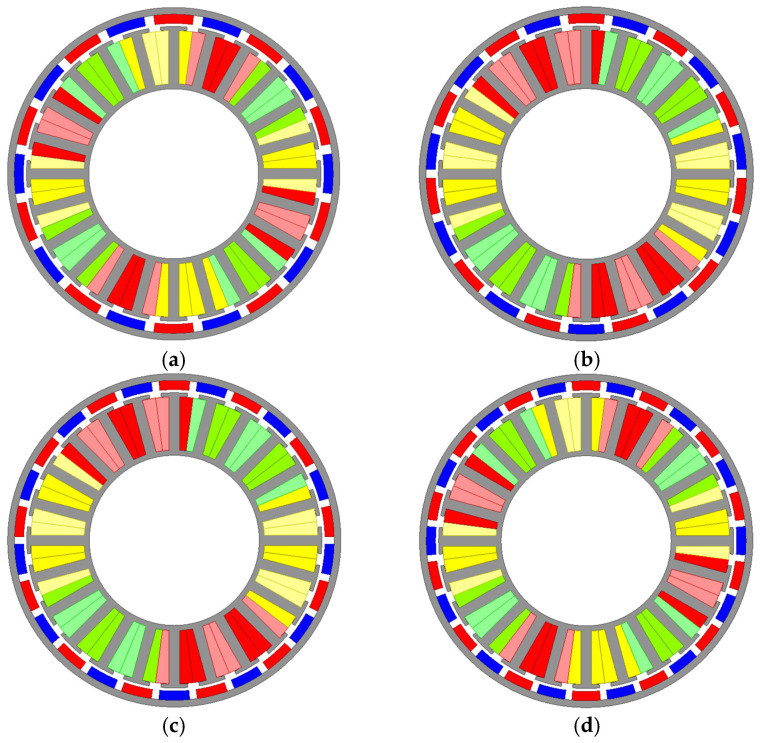
Motor topologies of 24-slot PM motors with different pole numbers: (**a**) 24-slot/20-pole; (**b**) 24-slot/22-pole; (**c**) 24-slot/26-pole; (**d**) 24-slot/28-pole.

Under the same stator parameters and pole arc coefficient, the electromagnetic performance comparison of 24-slot PM motors with different pole numbers is investigated in this section. Based on the design concept of fractional slot permanent magnet motors with non-overlapping concentrated windings, the 24-slot motor can choose 20, 22, 26, and 28 pole numbers to maximize the winding factor, i.e., 0.933, 0.949, 0.949, and 0.933, respectively. Figure 4 shows the motor topologies of 24-slot motors with different pole numbers. It can be seen that the winding distributions of motors with *N_s_* = 2*p* + 2 and *N_s_* = 2*p* − 2; *N_s_* = 2*p* + 4 and *N_s_* = 2*p* − 4 are the same, but that of motors with *N_s_* = 2*p* ± 2 and *N_s_* = 2*p* ± 4 are different. The comparison analysis includes no-load flux density distribution, air-gap flux density, back-EMF, cogging torque, electromagnetic torque, and torque–current curve.

### 4.1. No Load Condition

**Figure 5:**
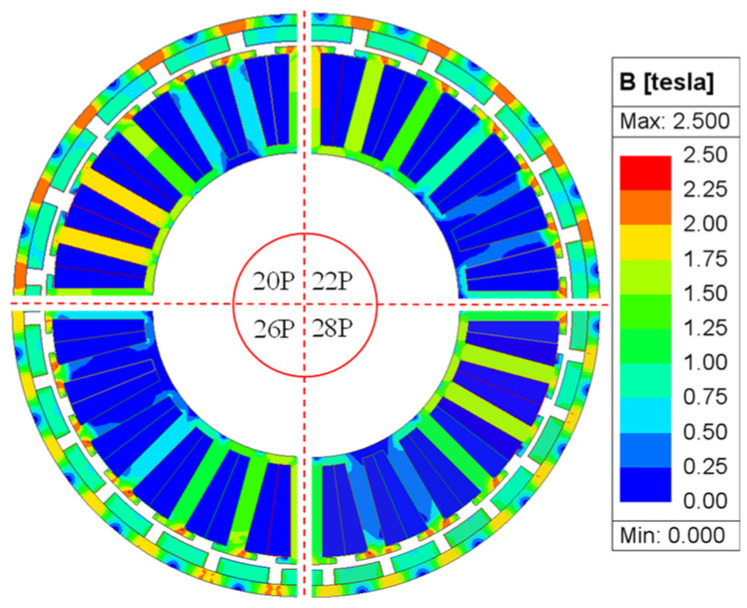
Flux density distributions of 24-slot PM motors with different pole numbers.

**Figure 6:**
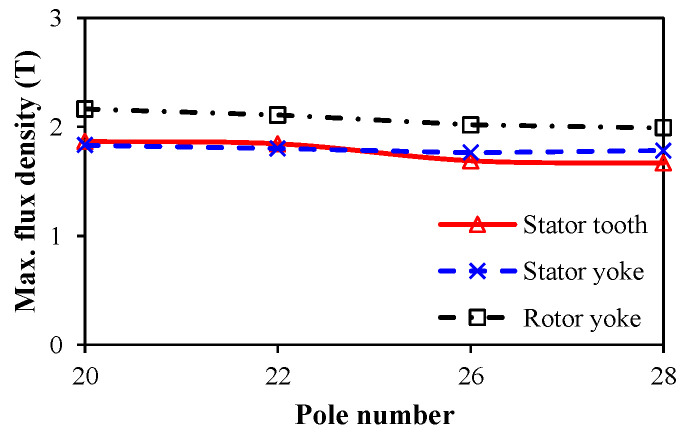
Variation in the maximum flux density in stator and rotor with pole number in the proposed 24-slot PM motor.

**Figure 7:**
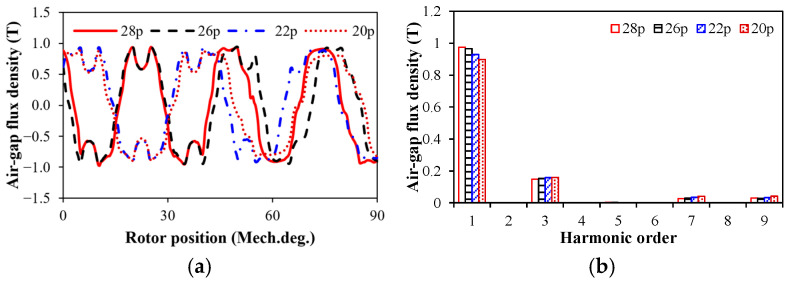
Open-circuit air-gap flux density waveforms and harmonics of the 24-slot PM motors with different pole numbers. (**a**) Open-circuit air-gap flux density. (**b**) Harmonic.

**Figure 8:**
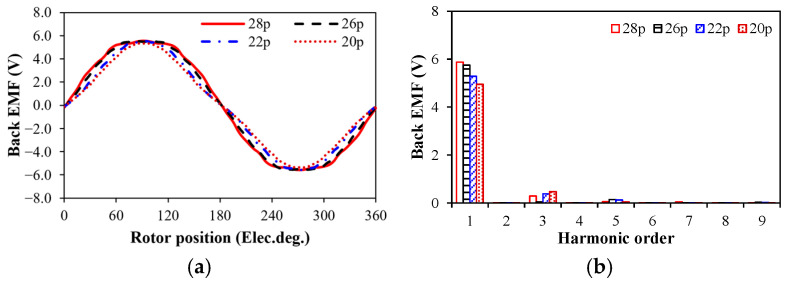
Back-EMF waveforms and harmonics of the 24-slot PM motors with different pole numbers at a rated speed of 700 r/min. (**a**) Back-EMFs. (**b**) Harmonic.

With the same stator structure, the different pole numbers will lead to different flux density distributions, as shown in Figure 5 and Figure 6. It can be seen that under the same rotor yoke thickness, the maximum flux densities of the stator and rotor core decrease with the increase in the pole number. Therefore, for the same flux density of the stator and rotor core, the higher pole number can reduce the stator tooth width and yoke thickness, achieving a larger slot area. Figure 7 shows the open-circuit air-gap flux densities of the 24-slot PM motors with different pole numbers. It can be seen that their air-gap flux density waves are almost sinusoidal and have the third, seventh, and ninth order harmonics due to the slot effect. In addition, the fundamental magnitude decreases with the decrease in the pole number. The phase A back-EMFs and harmonics of the 24-slot PM motors with different pole numbers are shown in Figure 8. As with the air-gap flux density, the fundamental magnitude of the back-EMF also decreases with the decrease in the pole number. However, the 24-slot/26-pole PM motor has almost no third-order harmonic.

**Figure 9:**
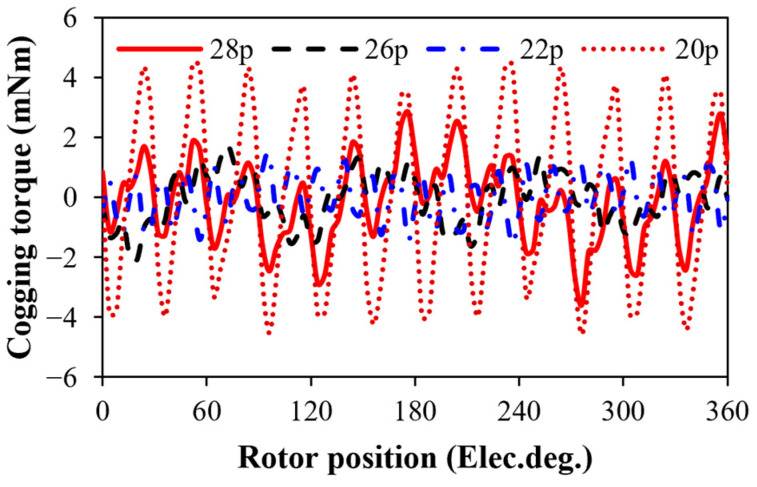
Cogging torque waveforms of the 24-slot PM motors with different pole numbers.

The greatest common divisor of the slot number (Z) and pole number (2p), i.e., GCD (Z, 2p), is an evaluation factor of the cogging torque. In general, the smaller the GCD (Z, 2p), the lower the peak value of the cogging torque, as shown in Table 3. Since the GCD (24, 20) and GCD (24, 28) are 4, the GCD (24, 22) and GCD (24, 26) are 2, the motors with 22-pole and 26-pole have relatively small peak values of cogging torque, but the motors with 20-pole and 28-pole have relatively large peak values of cogging torque, as shown in Figure 9. In addition to the evaluation factor of GCD (Z, 2p), the KL, which is the ratio of the slot number to the least common multiple of the slot and pole number, can also serve as a basis for assessing the cogging torque. Generally, the smaller the value of KL, the lower the cogging torque [29]. Therefore, the motor with 28 poles has a smaller peak value of cogging torque than the motor with 20 poles, in Figure 9.

### 4.2. Load Condition

**Figure 10:**
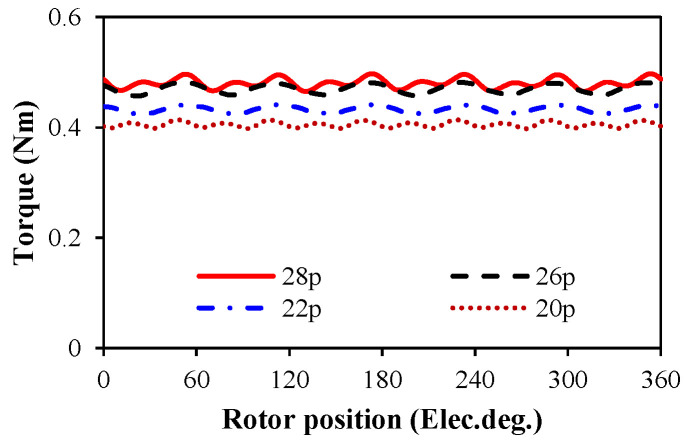
Torque waveforms of the 24-slot PM motors with different pole numbers.

With identical three-phase sinusoidal current excitation, the torque characteristics of the 24-slot PM motors with different pole numbers are investigated and presented in Figure 10. The electromagnetic torque waveforms are obtained through finite element analysis under the same current amplitude, ensuring a fair comparative assessment of the pole number effect on the torque generation capability. The analytical results demonstrate that the average torque exhibits a monotonic increasing trend with the augmentation of the pole number. Specifically, the 24-slot/28-pole PM motor achieves an average torque of 0.48 Nm, which represents a 20% enhancement compared with the 0.40 Nm output of the 24-slot/20-pole configuration. This torque improvement can be primarily attributed to the increased fundamental winding factor and the enhanced air-gap flux density associated with higher pole numbers, which collectively contribute to a more effective electromechanical energy conversion process. However, it is noteworthy that the 24-slot/28-pole PM motor simultaneously exhibits the most pronounced torque ripple among the investigated configurations, with the peak-to-peak ripple magnitude significantly exceeding those of the 20-pole and 22-pole counterparts. This phenomenon can be explained by the relatively severe magnetic saturation in the stator teeth and rotor yoke regions, which is an inherent consequence of the increased flux density levels required to achieve higher torque output. The localized saturation introduces harmonic distortions in the air-gap flux density distribution, thereby inducing undesirable torque pulsations that may deteriorate the motion control precision in high-performance servo applications.

**Figure 11:**
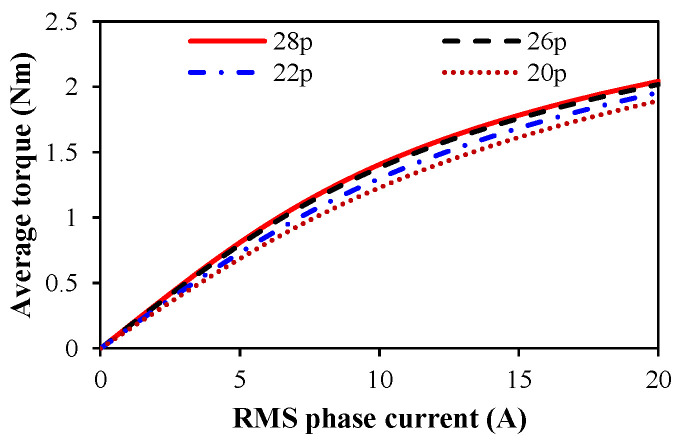
Variation in the average torque with the RMS phase current for the 24-slot PM motors with different pole numbers.

Furthermore, the torque–current characteristics of the four motors are examined by varying the RMS phase current from zero to the overload condition. As depicted in Figure 11, the average torque initially increases in an approximately linear manner with the escalation of the RMS phase current, indicating the unsaturated operating region where the electromagnetic torque is predominantly governed by the linear superposition of the permanent magnet flux linkage and the armature reaction field. Nevertheless, as the current continues to increase beyond a certain threshold, the slope of the torque–current curve gradually diminishes, manifesting the onset of magnetic saturation effects. The reduced incremental torque per unit current can be ascribed to the saturation-induced degradation of the winding inductance and the effective magnetomotive force, which collectively constrain the torque enhancement capability under high current excitation conditions. This nonlinear saturation characteristic is particularly evident in the rotor yoke in a 22-pole motor due to its inherently higher magnetic loading, implying that the overload torque capability is compromised.

**Figure 12:**
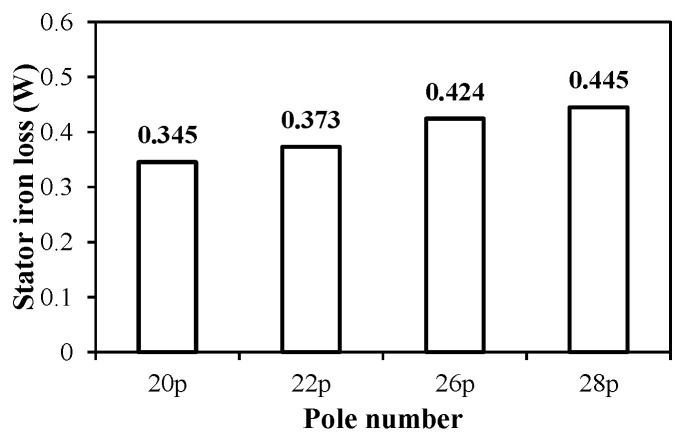
Variation in stator iron loss with the pole number.

At the rated operating speed of 700 r/min, the influence of the pole number on the stator iron loss characteristics is investigated and illustrated in Figure 12. The iron loss components, including the hysteresis loss and eddy current loss, are calculated based on the time-harmonic magnetic field distribution and the material-specific loss coefficients. Compared with the dominant copper loss of 17 W under rated conditions, the stator iron loss magnitude is relatively small and can be reasonably neglected in the preliminary thermal design and efficiency estimation. However, a quantitative comparison reveals that the ratio of the stator iron loss between the 20-pole and 28-pole configurations reaches approximately 30%, which is non-negligible from the perspective of loss distribution analysis and cooling system design. This discrepancy can be interpreted as follows: the increased pole number necessitates a higher stator core flux density to achieve the enhanced torque output, which in turn elevates the magnetic field variation rate and the associated core loss density. Consequently, although the absolute iron loss remains minor relative to the copper loss, the pole-number-dependent iron loss variation should be carefully considered in the comprehensive optimization of the motor efficiency, particularly for applications where continuous operation under varying speed conditions is required.

## 5. Electromagnetic Performance Comparison with Different Stators

In previous research in this paper, the influence of the pole number is investigated under the same stator structure. It mainly shows that the increased pole number can increase the air-gap flux density, and thus the average torque. However, with different pole numbers, the optimized motor design should be different. Therefore, the 24-slot PM motors with different pole numbers are optimized for the maximum torque. In this optimization process, the rotor yoke thickness, stator outer diameter, and the maximum flux density of the stator tooth are variables. The constraints include the rotor outer diameter, air-gap flux density, slot fill factor, and stator active length. By the automated optimization tools in Ansys Electronics Desktop 2023 R1, i.e., the Genetic Algorithm optimal method, four motors with different pole numbers are optimized for the maximum torque and the convergence criterion is 0.02. Table 4 shows the parameters of the optimized four PM motors with different pole numbers. It shows that with the decrease in the pole number:

- The optimal maximum stator flux density decreases;
- The optimal rotor yoke thickness decreases;
- The optimal stator outer diameter decreases, i.e., the optimal PM thickness decreases;
- The phase current decreases and current density increases.

It also shows that when the optimal maximum stator core flux density is 1.51 T, the rotor yoke thickness is 1.0 mm, and the stator outer diameter is 43.69 mm, the 24-slot/28-pole PM motor can achieve the maximum torque and relatively small torque ripple. In addition, the optimized 24-slot/28-pole PM motor has the smallest current density, which offers advantages of the low temperature rise under the same copper loss. Compared with the 24-slot/20-pole PM motor, the 28-pole motor has 12.7% higher torque under the same copper loss. From the optimization results presented in Table 4, it can be observed that when the maximum stator core flux density is constrained to 1.51 T, the rotor yoke thickness is optimized to 1.0 mm, and the stator outer diameter is determined as 43.69 mm, the 24-slot/28-pole PM motor achieves the highest output torque of 0.488 Nm with a relatively moderate torque ripple of 3.86%. Furthermore, the 24-slot/28-pole configuration exhibits the lowest current density of 17.90 A/mm^2^ among the four optimized motors, which offers a significant thermal advantage by ensuring a lower temperature rise under identical copper loss conditions. In quantitative terms, compared with the 24-slot/20-pole PM motor, the 28-pole motor delivers a 12.7% higher torque output under the same copper loss of 17 W, demonstrating superior electromagnetic utilization efficiency.

**Figure 13:**
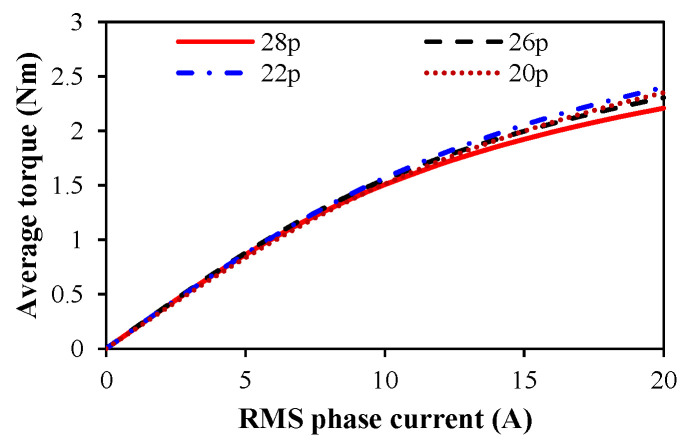
Variation in average torque with RMS phase current.

The overload capability characteristics of the optimized 24-slot PM motors with different pole numbers are further investigated, and the torque–current relationships are illustrated in Figure 13. The results indicate that the 22-pole motor exhibits the largest torque output at high current excitation levels, whereas the 28-pole motor demonstrates the smallest torque when the phase current exceeds 10 A. This phenomenon implies that the 24-slot/22-pole PM motor possesses the most favorable overload capability, particularly under extreme overload conditions exceeding 5 times the rated load. However, when the operating current is maintained below 10 A, i.e., below 3.5-time-load, the torque discrepancies among the four motors become negligible, suggesting comparable overload capabilities within the normal operating envelope.

Based on the comprehensive comparison of the electromagnetic performance metrics, including the output torque density, torque ripple suppression, thermal loading characteristics, and overload capability, the 24-slot/28-pole PM motor is identified as the most suitable candidate for humanoid robot joint applications. This configuration simultaneously achieves advantages in terms of high output torque, acceptable overload capability, and low thermal stress, which collectively satisfy the stringent requirements of compact size, high power density, and reliable continuous operation inherent to humanoid robotic actuators.

## 6. Thermal Analysis

**Figure 14:**
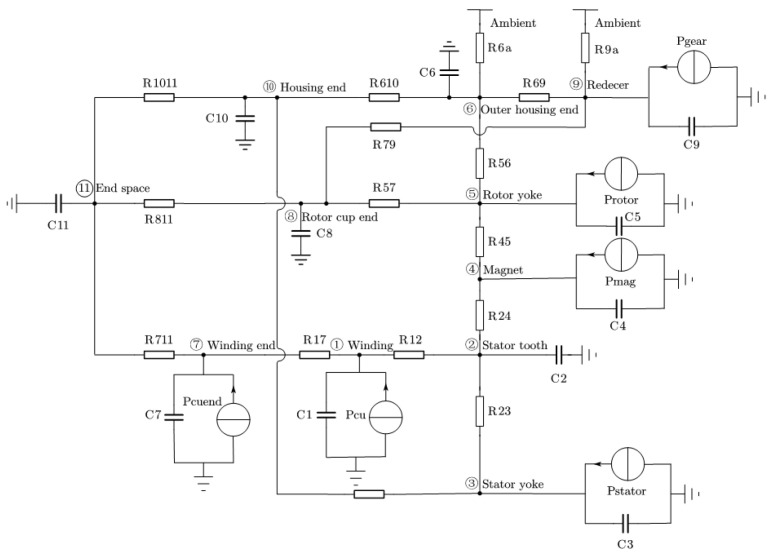
Two-dimensional lumped parameter thermal network model of the proposed motor.

For humanoid robot actuator applications, the thermal analysis of the proposed 24-slot/28-pole PM motor should be discussed considering the planetary gearbox by the lumped-parameter thermal network (LPTN) method. LPTN method is a modeling methodology for predicting motor temperature, which simplifies the heat conduction process into a discrete nodal network and analyzes it in the form of an equivalent circuit, thereby reducing the modeling complexity of intricate thermal systems [30,31]. Considering axial and radial heat transfer paths, a relatively complex LPTN model can improve the result accuracy [32,33]. Based on the structure of the actuator adopted in this paper, 11 temperature nodes are selected, including the winding, winding ends, stator tooth, stator yoke, magnet, rotor yoke, rotor cup end, housing end, outer housing end, reducer, and end space. The two-dimensional lumped parameter thermal network model of the proposed 24-slot/28-pole PM motor is shown in Figure 14. It is worth noting that the reducer is defined as a node connected with the ambient, outer housing end, and rotor cup end.

**Figure 15:**
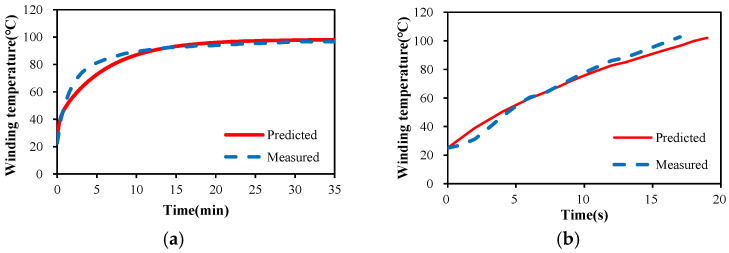
Winding temperature of the 24-slot/28-pole ERSPM under different conditions. (**a**) Rated condition (3.5 Nm@70 rpm). (**b**) 2-time overload condition (7.1 Nm@10 rpm).

Under the rated operating condition (3.5 Nm at 70 rpm), the predicted and measured temperatures of the windings are shown in Figure 15a. It shows that the winding temperature gradually increases and tends to stabilize at approximately 100 °C after 30 min of continuous operation, which is within the permissible thermal limit for Class F insulation systems. In addition, the predicted and measured results have good agreement, especially for the steady-state temperature and time. Figure 15b shows the winding temperature under the 2-time overload condition (7.1 Nm at 10 rpm). It can be seen that the winding temperature exhibits a rapid ascending trend and exceeds 100 °C merely after 16 s, subsequently triggering the over-temperature protection alarm. This phenomenon can be attributed to the significantly increased copper losses under high current density operation, which surpasses the heat dissipation capacity of the natural convection cooling scheme adopted in this prototype. It is worth noting that the predicted safe time is 19 s, which is slightly larger than the measured time, but has an acceptable agreement.

## 7. Experimental Validation

**Figure 16:**
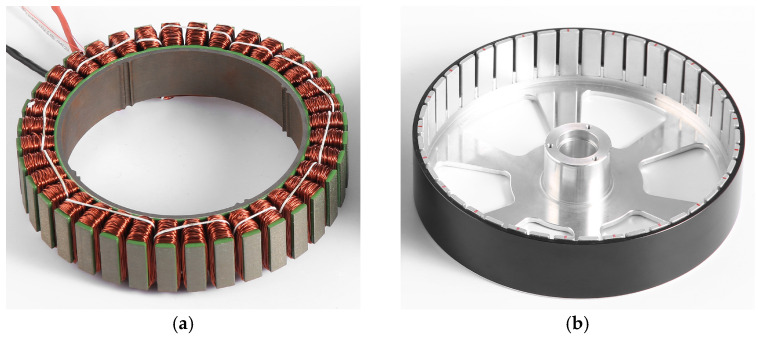
Prototype of 24-slot/28-pole ERSPM. (**a**) Wounded stator. (**b**) Cup-type rotor structure.

**Figure 17:**
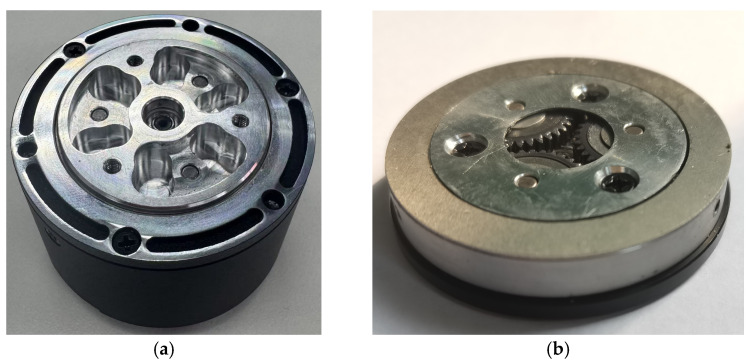
Prototype of actuator with 10:1 planetary gearbox. (**a**) Actuator. (**b**) Planetary gearbox.

**Figure 18:**
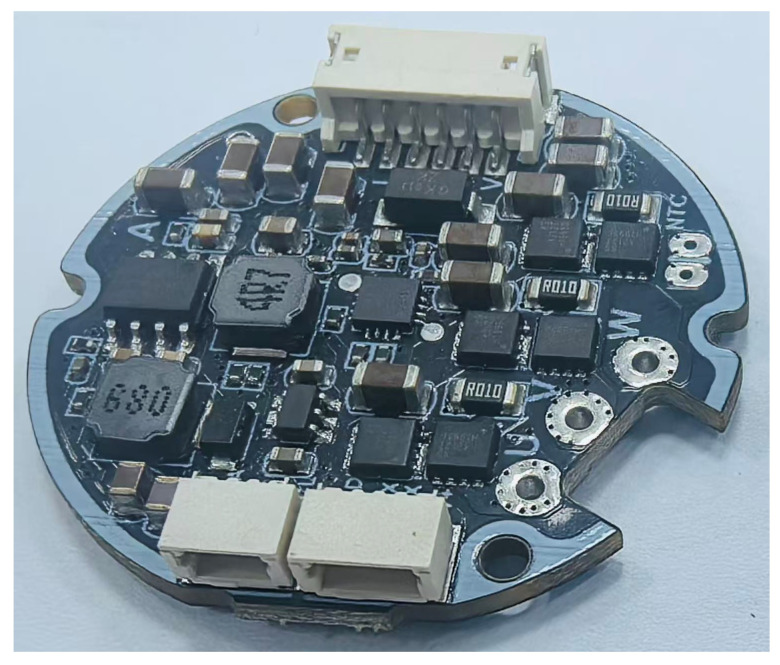
Actual driver hardware with 14-bit encoder.

**Figure 19:**
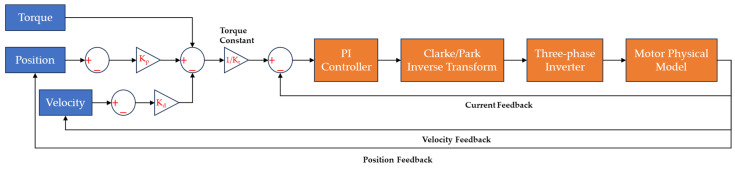
FOC three-loop control block diagram.

**Figure 20:**
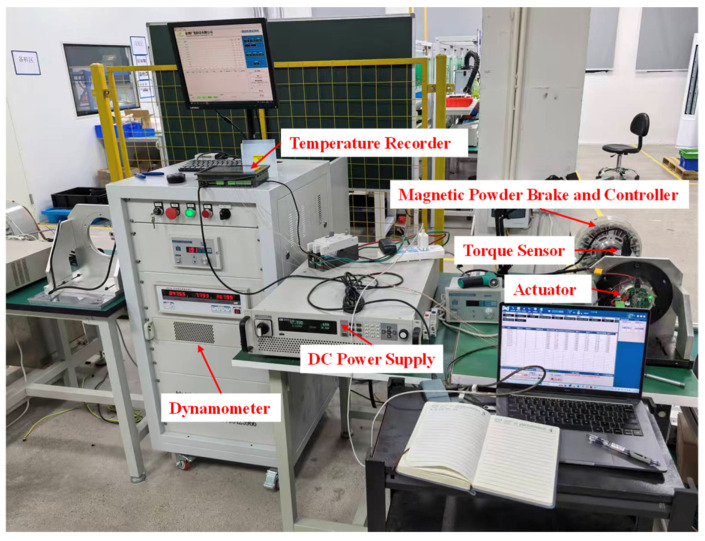
A comprehensive experimental test platform of humanoid robot actuators.

The optimized 24-slot/28-pole ERSPM has been manufactured to validate the proposed design methodology and finite element (FE) predictions. The wounded stator and rotor structures are illustrated in Figure 16, where the concentrated winding configuration and the surface-mounted permanent magnet arrangement can be clearly observed. It is worth noting that an NTC temperature sensor is located on the surface of the windings. The actuator prototype, integrated with a 10:1 planetary gearbox, is presented in Figure 17. Figure 18 and Figure 19 show the actual driver hardware with a 14-bit encoder and the FOC three-loop control block diagram, including current loop, speed loop, and position loop, respectively. A comprehensive experimental test platform is established to evaluate the electromagnetic and thermal performance of the manufactured prototype, as shown in Figure 20. The experimental setup comprises a dynamometer system, a temperature acquisition module, and a torque–speed measurement apparatus. The measured results are compared with the FE predictions to verify the accuracy of the electromagnetic design and thermal modeling.

**Figure 21:**
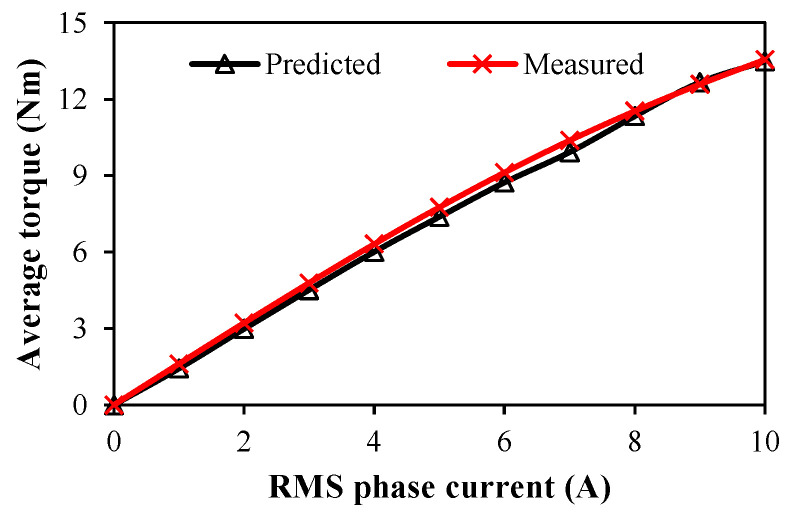
Variation in average torque with RMS phase current by FE predicted and measured.

**Figure 22:**
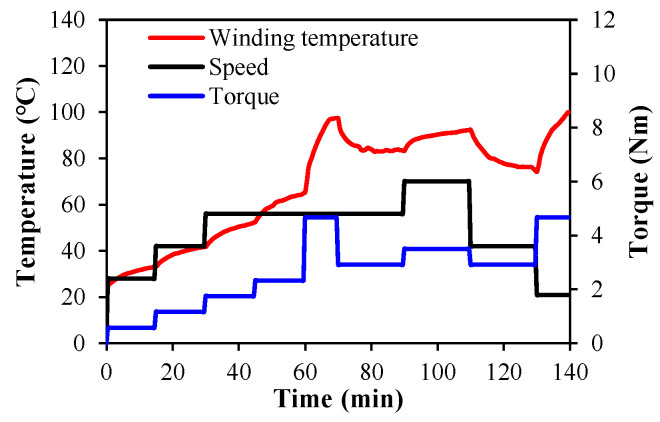
Measured winding temperature under dynamic loading.

Figure 21 presents the torque–current characteristics of the 24-slot/28-pole PM motor under various operating conditions. It is worth noting that the FE predicted torque values should be multiplied by a correction factor of 0.9 to account for the mechanical losses introduced by the planetary gearbox and the systematic measurement errors inherent in the experimental platform. Despite these practical non-idealities, the measured results demonstrate satisfactory agreement with the FE predicted results, thereby validating the effectiveness of the proposed motor design and optimization approach. Figure 22 shows the measured winding temperature under dynamic loading.

**Figure 23:**
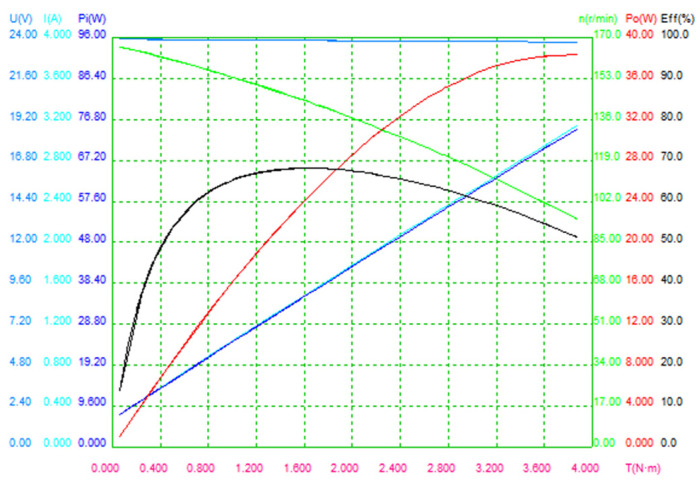
Efficiency curve of the 24-slot/28-pole ERSPM under different conditions and six values from left to right are input voltage(U), input current (I), input power (P_i_), speed (n), output power (P_o_), and efficiency (Eff).

The overall efficiency characteristics of the actuator are evaluated under varying load conditions, and the corresponding results are illustrated in Figure 23. The experimental results indicate that the highest efficiency reaches 68.3%, which encompasses the losses from the planetary gearbox, the motor itself, and the power electronic driver. The relatively moderate peak efficiency can be primarily attributed to the inherent mechanical losses of the 10:1 gearbox and the switching losses of the motor driver, which collectively impose constraints on the system-level efficiency optimization.

## 8. Conclusions

This paper compares the electromagnetic performance of the permanent magnet motors having similar slot and pole numbers. The influence of pole number on the optimization is mainly concentrated on the following points, such as the maximum stator flux density and split ratio. In addition, the 24-slot/28-pole ERSPM can achieve the largest output torque, smallest current density, and relatively high overload capacity. Therefore, this slot/pole number combination is selected for humanoid robot applications. Experimental measurements confirm agreement with FE predictions, and the winding temperature measurement under the rated and peak torque conditions shows the reliability of the proposed designed actuator.

## Figures and Tables

**Table 1:** Main parameters of the proposed motor.

Parameters	Values	Parameters	Values
Housing diameter (mm)	53	Stator inner diameter (mm)	>25
Housing thickness (mm)	1	Stator active length (mm)	20
Outer air-gap length (mm)	1	Rated speed (r/min)	70
Rotor outer diameter (mm)	49	Rated torque (Nm)	>3 Nm
Air-gap length (mm)	0.45	Peak torque (Nm)	>10 Nm

**Table 2:** Parameters of the optimized motor.

Parameters	Motor A	Motor B
Optimal goals	Max. torque	Max. torque density
Rotor outer diameter (mm)	49	
Rotor yoke thickness (mm)	1.0	
Air-gap length (mm)	0.45	
Magnet thickness (mm)	1.35	1.85
Stator outer diameter (mm)	43.4	42.4
Stator tooth width (mm)	1.55	1.63
Stator yoke thickness (mm)	0.774	0.815
Stator inner diameter (mm)	25	
Stator active length (mm)	10	
Rated speed (r/min)	700	
Copper loss (W)	17	
RMS phase current (A)	2.87	2.70
Torque (Nm)	0.481	0.461
ΔT (%)	(0.481–0.461)/0.461 = 4.3%	
Weight (g)	113.10	110.40
Torque density (Nm/kg)	4.253	4.172
ΔTD (%)	(4.253–4.172)/4.172 = 1.9%	
PM volume (mm^3^)	1548.87	2099.275
PM utilization (Nm/m^3^)	300.22	219.60
ΔPMU (%)	(300.22–219.6)/300.22 = 26.8%	
Split ratio	0.886	0.865

**Table 3:** Evaluation factors of the cogging torque.

24-Slot	28p	26p	22p	20p
GCD (Z, 2p)	4	2	2	4
Z/LCM (Z, 2p)	24/168 = 0.14	24/312 = 0.08	24/264 = 0.09	24/120 = 0.2
Peak cogging torque (mNm)	2.84	1.75	1.46	4.49

**Table 4:** Parameters of the optimized four motors.

Parameters	28p	26p	22p	20p
Maximum stator core flux density (T) [1.2–2]	1.51	1.46	1.36	1.33
Rotor yoke thickness (mm) [0.8–1.5]	1.00	1.03	1.17	1.22
Stator outer diameter (mm) [40–44]	43.69	43.47	43.15	43.15
PM thickness (mm)	1.205	1.285	1.305	1.255
Torque (Nm)	0.488	0.482	0.455	0.433
Torque ripple (%)	3.86	4.27	3.75	5.21
Copper loss (W)	17			
RMS phase current (A)	2.74	2.66	2.54	2.53
Torque factor (Nm/A)	0.1781	0.1812	0.1791	0.1712
Current density (A/mm^2^)	17.90	18.36	19.11	19.20

## Data Availability

No new data were created or analyzed in this study.
